# Predicting depressed and elevated mood symptomatology in bipolar disorder using brain functional connectomes

**DOI:** 10.1017/S003329172300003X

**Published:** 2023-03-09

**Authors:** Anjali Sankar, Xilin Shen, Lejla Colic, Danielle A. Goldman, Luca M. Villa, Jihoon A. Kim, Brian Pittman, Dustin Scheinost, R. Todd Constable, Hilary P. Blumberg

**Affiliations:** 1 Department of Psychiatry, Yale School of Medicine, New Haven, CT, USA; 2 Neurobiology Research Unit, Copenhagen University Hospital, Rigshospitalet, Copenhagen, Denmark; 3 Department of Radiology and Biomedical Imaging, Yale School of Medicine, New Haven, CT, USA; 4 Department of Psychiatry and Psychotherapy, Jena University Hospital, Jena, Germany; 5 German Center for Mental Health, Halle-Jena-Magdeburg, Magdeburg, Germany; 6 Interdepartmental Neuroscience Program, Yale School of Medicine, New Haven, CT, USA; 7 Department of Psychiatry, University of Oxford, Oxford, UK; 8 Child Study Center, Yale School of Medicine, New Haven, CT, USA

**Keywords:** Bipolar disorder, CPM, functional magnetic resonance imaging, symptom severity

## Abstract

**Background.:**

The study is aimed to identify brain functional connectomes predictive of depressed and elevated mood symptomatology in individuals with bipolar disorder (BD) using the machine learning approach Connectome-based Predictive Modeling (CPM).

**Methods.:**

Functional magnetic resonance imaging data were obtained from 81 adults with BD while they performed an emotion processing task. CPM with 5000 permutations of leave-one-out cross-validation was applied to identify functional connectomes predictive of depressed and elevated mood symptom scores on the Hamilton Depression and Young Mania rating scales. The predictive ability of the identified connectomes was tested in an independent sample of 43 adults with BD.

**Results.:**

CPM predicted the severity of depressed [concordance between actual and predicted values (*r* = 0.23, *p*_perm (permutation test)_ = 0.031) and elevated (*r* = 0.27, *p*_perm_ = 0.01) mood. Functional connectivity of left dorsolateral prefrontal cortex and supplementary motor area nodes, with inter- and intra-hemispheric connections to other anterior and posterior cortical, limbic, motor, and cerebellar regions, predicted depressed mood severity. Connectivity of left fusiform and right visual association area nodes with inter- and intra-hemispheric connections to the motor, insular, limbic, and posterior cortices predicted elevated mood severity. These networks were predictive of mood symptomatology in the independent sample (*r* ≥ 0.45, *p* = 0.002).

**Conclusions.:**

This study identified distributed functional connectomes predictive of depressed and elevated mood severity in BD. Connectomes subserving emotional, cognitive, and psychomotor control predicted depressed mood severity, while those subserving emotional and social perceptual functions predicted elevated mood severity. Identification of these connectome networks may help inform the development of targeted treatments for mood symptoms.

## Introduction

Depressed and elevated mood symptom severity underlie the suffering associated with bipolar disorder (BD) and are among the strongest clinical predictors of functional impairment and disability in the disorder (Judd et al., 2005). Furthermore, mood symptoms contribute to the high risk for suicide in the disorder. Elucidation of the brain functional disturbances that contribute to mood symptoms in individuals with BD could advance our understanding of the pathophysiology of the disorder and enable the development of more targeted therapeutic approaches.

Previous functional magnetic resonance imaging (fMRI) studies investigating brain functional disturbances associated with mood states often used categorical determinations of participants meeting criteria for depressed or elevated episodes. Only to a lesser extent was mood assessed dimensionally by severity scales even though this approach has the benefit of providing information regarding symptom severity (Sankar et al., 2020). Findings were commonly observed in the prefrontal cortex (PFC), with patterns of dysfunction suggested to vary by mood state in their relative hemispheric and ventral v. dorsal distribution. Depressed episodes of BD, the most common and studied of the acute BD episode types, were associated with dysfunction in the left PFC and dorsolateral PFC (dlPFC) areas (Altshuler et al., 2008; Goldman et al., 2022). Dysfunctions in the dlPFC have also been associated with depressed symptom severity (Bertocci et al., 2020; Brooks et al., 2009; Fernández-Corcuera et al., 2013; Ketter et al., 2001; Koenigs & Grafman, 2009). Studies of elevated mood episodes, albeit fewer, suggest dysfunction particularly in the right hemisphere and in ventral PFC (vPFC) areas (Blond & Blumberg, 2010; Blumberg et al., 1999, 2003; Elliott et al., 2004; Liu et al., 2012; Strakowski et al., 2011). Although there is a convergence of finding in the PFC, neuroimaging investigations of mood states of BD have increasingly suggested that dysfunction at the system level, rather than solely in isolated brain regions, contributes to BD mood episodes and symptom severity. For example, in elevated mood states, in addition to findings in the right vPFC, there is also some evidence for dysfunction in larger scale networks comprising limbic, predominantly amygdala (Cerullo et al., 2012; Li et al., 2015), and temporal, particularly fusiform area (Adleman et al., 2013a, 2013b; Strakowski et al., 2011), parietal (Öngür et al., 2010; Zhang et al., 2020), and occipital cortical regions (Manelis et al., 2019). In addition to distributed intrahemispheric findings, reports also suggest disrupted inter-hemispheric connectivity in BD (Blumberg et al., 2003; Liu et al., 2012).

Findings from the above fMRI studies are based on examining average group-level differences or correlations. The effect sizes from such traditional methods are typically not high enough to make individual-level inferences (Abi-Dargham & Horga, 2016). Furthermore, for neuroimaging findings to be defined as biomarkers, they need to be replicable when tested in an independent dataset (Orru, Pettersson-Yeo, Marquand, Sartori, & Mechelli, 2012; Orrù, Monaro, Conversano, Gemignani, & Sartori, 2020). Machine learning approaches, such as the recently developed connectome-based predictive modeling (CPM) method (Shen et al., 2017), are beneficial as they allow the prediction of behavior or symptom severity at the individual level by determining the most optimal predictive model using multivariate brain metrics and involve tests of whether the same model can predict the same behavior in an independent sample. Thus, machine learning based analyses (e.g. CPM) permit advances toward providing foresight with clinical relevance by predicting behavior of individual subjects (Hahn, Nierenberg, & Whitfield-Gabrieli, 2017; Walter et al., 2019).

CPM has been previously used to identify functional brain connectivity matrices, referred to as connectome ‘fingerprints,’ that predict individual differences in traits and behaviors such as fluid (Finn et al., 2015) and sustained (Rosenberg et al., 2016; Rosenberg, Hsu, Scheinost, Todd Constable, & Chun, 2018) intelligence, and neuroticism and extraversion (Hsu, Rosenberg, Scheinost, Constable, & Chun, 2018). More recently, CPM was used to identify connectome fingerprints of anxiety severity (Wang et al., 2021), and treatment response in major depressive (Fan et al., 2020; Ju et al., 2020) and substance use (Yip, Scheinost, Potenza, & Carroll, 2019) disorders. Typically, resting state data have been used in fMRI studies to make individual-level predictions about network alterations in psychiatric disorders. However, recent evidence suggests that salient tasks can modulate brain functional networks and amplify disorder-relevant individual differences in patterns of functional connectivity (Barron et al., 2019; Greene, Gao, Scheinost, & Constable, 2018), akin to the use of stress tests to identify cardiac dysfunction. Therefore, prediction models built using salient task data tend to outperform those built with resting state data (Greene et al., 2018). In the present fMRI study, participants performed a task in which they processed emotional face stimuli salient to the emotional dysfunction characteristic of BD and shown repeatedly to elicit brain differences between individuals with BD compared to healthy control individuals (Blumberg et al., 2003; Johnston et al., 2017; Liu et al., 2012; Wang, Bobrow, Liu, Spencer, & Blumberg, 2012). In this study, CPM was applied to a dataset of adults with BD and further tested on an independent dataset of adults with BD to examine if (a) models built on the emotional task data can predict severity of depressed and elevated mood symptomatology in BD at the individual-level, and (b) findings replicate in an independent dataset of adults with BD.

To our knowledge, this is the first study to use a machine learning approach to predict individual-level depressed and elevated mood symptomatology in BD. Based on previous literature exploring group-level differences in brain function, we expected that whole-brain, intra- and inter-hemispheric functional connectivity alterations in a left dlPFC system would predict individual differences in depressed mood symptom severity (Altshuler et al., 2008; Brooks et al., 2009; Fernández-Corcuera et al., 2013; Ketter et al., 2001; Luo et al., 2018) and in a right vPFC system would predict individual differences in elevated mood symptom severity (Blumberg et al., 2003; Liu et al., 2012).

## Methods

### Participants in the training dataset

Eighty-one participants met criteria for BD according to the Diagnostic and Statistical Manual of Mental Disorders (Fourth ed. Text Revision; DSM-IV-TR) [ages 18–55 years, mean age ± standard deviation (S.D.) = 29.3 ± 11.1 years; 63.0% female]. The presence or absence of Axis I diagnoses and mood state for all participants were confirmed with the Structured Clinical Interview for DSM-IV Diagnosis (SCID) (First, 2014). Two-thirds of the participants were on psychotropic medications at the time of scan. Exclusion criteria were major medical disorders (except treated hypothyroidism), central nervous system conditions including a history of loss of consciousness ≥5 min, substance or alcohol abuse or dependence within three months of MRI scanning, and MRI contraindications. The demographic and clinical characteristics of the sample are detailed in the online Supplementary section (Table S1). Written informed consent was obtained from all participants. The authors assert that all procedures were conducted in accordance with the Yale School of Medicine (SOM) Human Investigation Committee institutional review board.

Mood symptom severity of all the participants was assessed using the Hamilton Depression Rating Scale 29-item version (HDRS-29) (Williams, Link, Rosenthal, & Terman, 1988) (mean ± S.D. = 12.3 ± 10.1; range:0–40) and the Young Mania Rating Scale (YMRS) (Young, Biggs, Ziegler, & Meyer, 1978) (mean ± S.D. = 6.5 ± 6.1; range:0–23). However, as the HDRS-29 scale is multifactorial, and measures more than one symptom cluster which may have different underlying neurobiological bases, a depressed mood score (HDRS-5) was calculated, as done in our previous work (11), by summing five items from the HDRS (HDRS-5) that showed the highest loading for depression (i.e. depressed mood, work and interests, guilt, psychomotor retardation, and suicide (12) (HDRS-5 mean ± S.D. = 3.4 ± 3.4; range:0–13). The YMRS on the other hand, has been found to have higher validity when used in the one-factor format (i.e. using total scores) (Serrano, Ezpeleta, Alda, Matalí, & San, 2011; Youngstrom, Kmett Danielson, Findling, Gracious, & Calabrese, 2002). Furthermore, structures of this scale have not been well-established as they have been tested only using small (e.g. *n* = 17) sample sizes (Double, 1990). Therefore, in the present study, the YMRS scale, with the total score obtained by summing all the eleven items of the scale, was used to predict elevated mood severity.

### MRI data acquisition

A 3-Tesla Trio scanner was used for MR scanning (Siemens, Erlangen, Germany). After a localizing scan, a high-resolution 3-dimensional volume was obtained using magnetization-prepared rapid gradient-echo (MPRAGE) sequence (repetition time (TR): 1500 ms; echo time (TE): 2.77 ms; flip angle (FA): 15°; matrix: 256 × 256; field of view (FOV): 256 mm × 256 mm; slice thickness, 1.0 mm without gap; 160 contiguous slices). Two-dimensional T1-weighted images were also obtained (TR: 300 ms; TE: 2.47 ms; FA: 60°; matrix: 256 × 256; FOV: 256 × 256 mm^2^; slice thickness: 3 mm without gap). FMRI data were collected with a T2*-weighted single-shot echo planar imaging sequence with Blood Oxygen Level-Dependent contrast, aligned with the anterior commissure-posterior commissure plane (TR: 2000 ms; TE: 25 ms; FA: 80°; matrix: 64 × 64; FOV: 240 mm × 240 mm; slice thickness: 3.0 mm without gap; 32 contiguous slices). FMRI data were obtained while participants performed an emotional face gender-labeling task, as reported previously, in which they viewed faces from the Ekman series depicting happy, fearful, or neutral expressions for 2 s (sec), separated by 4–12 s periods viewing a fixation cross-hair, and pressed a button to indicate whether the face belonged to a male or a female (Johnston et al., 2017; Wang et al., 2012). Participants performed four consecutive runs of the task, each lasting 4 min (min) and 50 s. FMRI data collection time was 19 min 20 s.

### Preprocessing

The first four volumes of each functional run were discarded to allow for hemodynamic steady state. Slice timing and motion correction were performed; all participants had an average framewise displacement of <0.2 mm. Images were iteratively smoothed with a Gaussian filter of 6 mm full-width at half-maximum (FWHM) using AFNI’s 3dBlurToFWHM (afni.nimh.nih.gov/afni/) (Scheinost, Papademetris, & Constable, 2014). Further preprocessing steps were performed using BioImage Suite (bioimagesuite.org). Covariates of no interest were regressed from the data, including linear and quadratic drifts, mean cerebrospinal fluid, mean white matter signal, mean gray matter signal, and 24-parameter motion variables (including six rigid-body motion parameters, six temporal derivatives, and their squares). Functional connectivity was calculated based on the ‘raw’ task time courses, i.e., without regressing out task-evoked activity. The data were temporally smoothed with a Gaussian filter (~cut-off frequency = 0.12 Hz). Finally, data from the four runs were variance normalized and concatenated for each participant. To allow images to be compared across participants, all images were warped into MNI (Montreal Neurological Institute) space using a series of linear and non-linear registrations. The fMRI data were linearly registered to the 2-dimensional anatomical images. The 2-dimensional anatomical images were linearly registered to the 3-dimensional MPRAGE images. The 3-dimensional MPRAGE images were then non-linearly registered to the MNI template using a previously validated algorithm (Scheinost et al., 2017). All transformation pairs were calculated independently and then combined into a single transformation by which single participant images (i.e. functional, 2-dimensional anatomical, and 3-dimensional MPRAGE images of a participant) were warped into MNI space. This single transformation was performed to reduce interpolation error and was done using BioImage Suite.

### Connectivity matrices

Whole brain functional connectivity was assessed using methods described previously (Finn et al., 2015; Shen et al., 2017). In brief, network nodes were defined using the Shen 368-node functional brain atlas (Horien, Shen, Scheinost, & Constable, 2019), which includes the cortex, subcortex, cerebellum, and brainstem. For the cortex, Shen et al., applied a group-wise parcellation algorithm (Shen, Papademetris, & Constable, 2010) and obtained 164 nodes in the left hemisphere and 163 nodes in the right hemisphere. This functional parcellation of the cortex was computed based on resting-state BOLD data from 120 participants. For the subcortical area, Shen et al. adopted anatomical definitions of subcortical structures (Lacadie, Fulbright, Rajeevan, Constable, & Papademetris, 2008) with seven nodes in each of the left and right subcortical regions. For the cerebellum, they adapted the 17 network definition proposed by Buckner et al. (Buckner, Krienen, Castellanos, Diaz, & Yeo, 2011) eliminating some nodes of small size, leaving 13 nodes in each of the left and right cerebellum. They also included one node for the brain stem area. For each participant, the mean time course for each of the 368 nodes was extracted to compute node-by-node pairwise Pearson’s correlation coefficients. The r values were transformed using Fisher’s *z* transformation resulting in a symmetric 368 by 368 connectivity matrix of correlation values representing edges. The edges can be visualized as connections between nodes that are assigned to one of ten bilateral macroscale brain regions of the cortex, subcortex, cerebellum, or brainstem. One subject was excluded from the analysis due to its distribution of edge weights being an outlier when compared to the rest of the participants.

### Connectome-based Predictive Modeling (CPM)

CPM was conducted using previously validated custom MATLAB scripts (Shen et al., 2017). CPM uses connectivity matrices (edges) and clinical data as input variables to generate a predictive model of the clinical data from the edges. Edges and clinical data from the training data set are correlated to identify positive and negative predictive networks. The most relevant edges are selected for further analysis based on the significance of the linear association between edge and clinical data. Consistent with previous CPM studies, a threshold of *p* < 0.001 was set to select edges (Cai, Zhu, & Yu, 2020; Song et al., 2020). Positive networks are comprised of increased edge weights (increased connectivity) associated with the clinical scores, and negative networks of decreased edge weights (decreased connectivity) associated with clinical scores. Together, the positive and negative networks make up the combined or the overall network model. Single-subject summary statistics are then calculated by separately summing the significant edge weights in the positive and negative networks which are then used to determine coefficients of the linear model relating network strength with the clinical data. The resulting polynomial coefficients, which include the slope and intercept of the linear model, are then applied to the test data set to predict clinical scores (cross-validation approach). In this study, a leave-one-out cross-validation (LOOCV) was used. The common edges across the LOOCV iterations are used for model interpretation.

### Permutation testing

When using LOOCV, analyses in the leave-one-out folds are not wholly independent, and the number of degrees of freedom is thus overestimated for parametric *p* values based on correlation. Permutation testing was therefore performed instead of parametric testing. To generate null distributions for significance testing, the correspondence between clinical scores and edges was randomly shuffled 5000 times and the CPM analysis was rerun with the shuffled data. The *p* values for leave-one-out predictions ( *p*_perm_) were calculated by determining the percentage of permutations that generated correlation coefficients larger than the correlation coefficients from the original (unshuffled) leave-one-out predictions.

### Predictor and control variables

Clinical data that were used as predictor variables in this study were HDRS-5 and YMRS total scores. Exploratory analysis using total HDRS scores (HDRS-29) as predictor variables was also performed. Partial Pearson correlations of predicted and observed scores were calculated controlling for age and gender. It was confirmed that the average framewise displacement did not correlate with the predictor variables in the sample (i.e. HDRS-5 and YMRS scores, *p* > 0.13). Furthermore, the predictor variables were not correlated (*p* = 0.33) in the current sample.

### Out-of-sample validation

To determine the generalizability of the findings, the resultant CPM models from the training dataset were tested in an independent dataset that had no overlapping participants with the training dataset. The participants in the validation sample consisted of 43 right-handed individuals with BD [mean ± S.D. (years) = 26.8 ± 8.9; range:18–57 years; 60.5% female]. Scanning for 34 participants was performed on the same 3-Tesla Trio scanner and with the same parameters as the training dataset. Scanning for nine participants was performed using a 3-Tesla Siemens PRISMA scanner with parameters: 3-dimensional MPRAGE (TR = 2500 ms, TE = 2.81 ms, matrix = 256 × 256, FOV = 256 mm × 256 mm, 176 one-mm slices without gap), 2-dimensional T1 image (TR: 400 ms; TE: 2.61 ms; FA: 60°; matrix: 192 × 192; FOV: 220 mm × 220 mm; slice thickness: 2 mm without gap) and fMRI task (TR: 1000 ms, TE: 29.6 ms, matrix = 110 × 110, FOV: 1980mm × 1980 mm, flip angle 60, 72.2 mm slices).

There were no significant differences in age (Independent Samples Mann–Whitney *U* Test; *U* = 1610.5, *N* = 127, *p* = 0.3) or gender (Chi-square Test; *χ*^2^(2127) = 0.03, *p* = 0.9) between the training and the out-of-sample validation datasets. Two participants in the out-of-sample group did not have YMRS scores; hence, predictions of YMRS scores were performed in 41 participants. Of the 43 participants, 11 had current substance abuse (2 alcohol, 1 cannabis, 1 stimulant) and dependence (4 cannabis, 3 alcohol), and 14 had fewer than four (range: 1–3) fMRI task runs. As above, individual subject summary scores were calculated by separately summing the significant edge weights in the positive and negative networks which were extracted from functional connectivity matrices for all participants and then entered into regression analyses with HDRS-5 and YMRS scores.

## Results

### Prediction of depressed mood severity

**Fig. 1.**
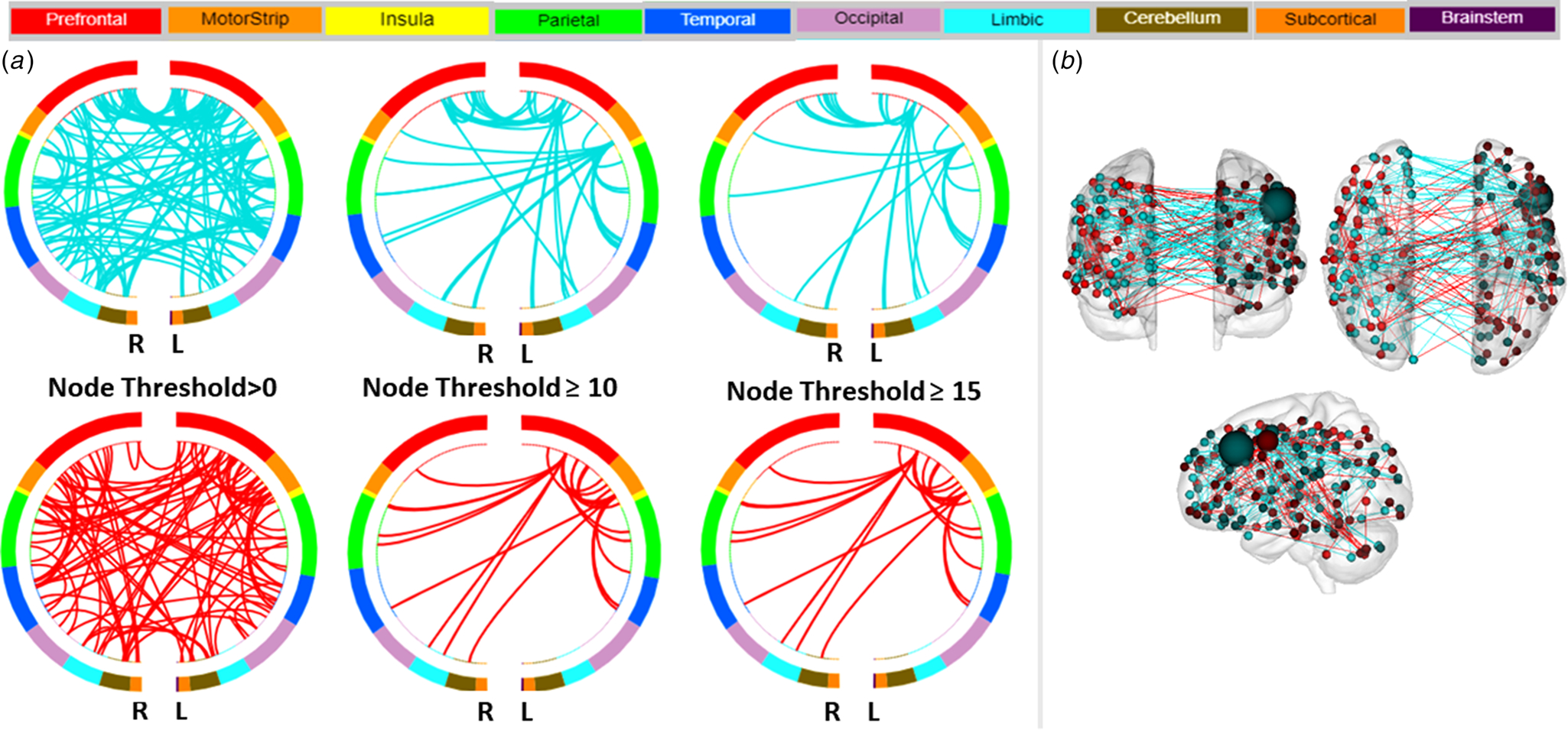
Negative and Positive Networks Predicting Severity of Depressed Mood using Connectome-based Predictive Modeling (CPM). (a) Circle plots in which nodes are assigned to one of ten bilateral macroscale brain regions. Negative and positive edges (or connections between nodes) are depicted on separate plots at different thresholds for visualization. Threshold values indicate the minimum number of connections emanating from a node. For the negative network (connections depicted in blue), decreased edge weights (i.e. decreased functional connectivity) predict severity of depressed mood, based on five items on the Hamilton Depression Rating Scale that showed the highest loading for depression (i.e. depressed mood, work and interests, guilt, psychomotor retardation, and suicide). For the positive network (in red), increased edge weights (i.e. increased functional connectivity) predict severity of depressed mood. R, right hemisphere; L, left hemisphere. (b) Glass brain depicting strength of negative and positive networks, depicted in blue and red respectively. Each node is represented as a sphere; the size of the sphere indicates the number of edges emanating from that node. The highest degree node, i.e., nodes with the most connections (edges), contributing to the prediction of depressed mood severity was a node located in the left dorsolateral prefrontal cortex in the negative network.

The overall CPM model predicted the severity of depressed mood (HDRS-5) (combined positive and negative networks: *r* = 0.23, root mean square error (RMSE) = 3.5, *p*_perm_ = 0.031; online Supplementary Fig. S1). Investigation of the positive and negative networks separately showed a greater contribution for the prediction of depressed mood from the negative network (*r* = 0.36, RMSE = 3.3, *p*_perm_ < 0.001). Figure 1a, b displays negative and positive ‘depressed mood’ networks, defined by the macroscale regions, that significantly predicted depressed mood severity. The high degree nodes, i.e., nodes with the most connections (edges), in the negative network included a left PFC node located in the dlPFC and a left motor node located in the supplementary motor area (SMA). In predicting depressed mood severity, the left dlPFC showed lower inter-hemispheric connections to PFC nodes (right dlPFC and rostral PFC), lower intra-hemispheric connections to other PFC (left medial orbitofrontal cortex), limbic (left ventral anterior cingulate cortex, vACC, and dorsal ACC, dACC) and temporal (left inferior temporal gyrus) nodes, and lower inter- and intra-hemispheric connections to cerebellum nodes. The left SMA node showed lower inter-hemisphere connections to a limbic node (right amygdala), lower intra-hemispheric connections to other limbic (left dorsal posterior cingulate cortex, dPCC), and temporal (left superior temporal gyrus) nodes, and lower inter- and intra-hemispheric connections to parietal (bilateral supramarginal gyrus) and motor (bilateral primary motor area) nodes. The high degree nodes in the positive network also included left PFC and motor nodes, and as expected, the regions of their connections differed from those of the negative network. The left prefrontal node (dlPFC) showed increased inter-hemispheric connections to prefrontal (right ventrolateral PFC), insular, and limbic (including right vACC and dPCC) nodes, increased intra-hemispheric connections to motor node (left SMA), and increased inter- and intra-hemispheric connections to parietal nodes (bilateral supramarginal gyrus). The left motor node (SMA) showed increased inter-hemispheric connections to the cerebellar node, increased intra-hemispheric connections to prefrontal (including left dlPFC and rostral PFC) nodes, and increased inter- and intra-hemispheric connections to temporal nodes (bilateral fusiform). The high degree nodes and their connections in the negative and positive network, and the respective macroscale regions they are assigned to, are detailed in Table 1. Sensitivity analysis when the model was also controlled for YMRS severity showed the overall CPM model still predicted HDRS-5 severity (combined positive and negative networks: *r* = 0.22, *p*_perm_ = 0.03); furthermore, the analysis showed no additional high degree nodes.

Exploratory investigation with the HDRS-29 scores revealed that the overall CPM model predicted total HDRS-29 scores (combined positive and negative networks: *r* = 0.27, *p*_perm_ = 0.01). The high degree node in the left dlPFC in the negative network that contributed to prediction of HDRS-5 scores was also a high degree node that contributed to the prediction of HDRS-29 scores. Other high degree nodes contributing to HDRS-29 score prediction in the negative and positive networks included a node in the right dlPFC and another in the right cerebellum. The high degree nodes and their connections in the negative and positive network, and the respective macroscale regions they are assigned to, are detailed in the online Supplementary section (Table S2).

### Prediction of elevated mood severity

**Fig. 2.**
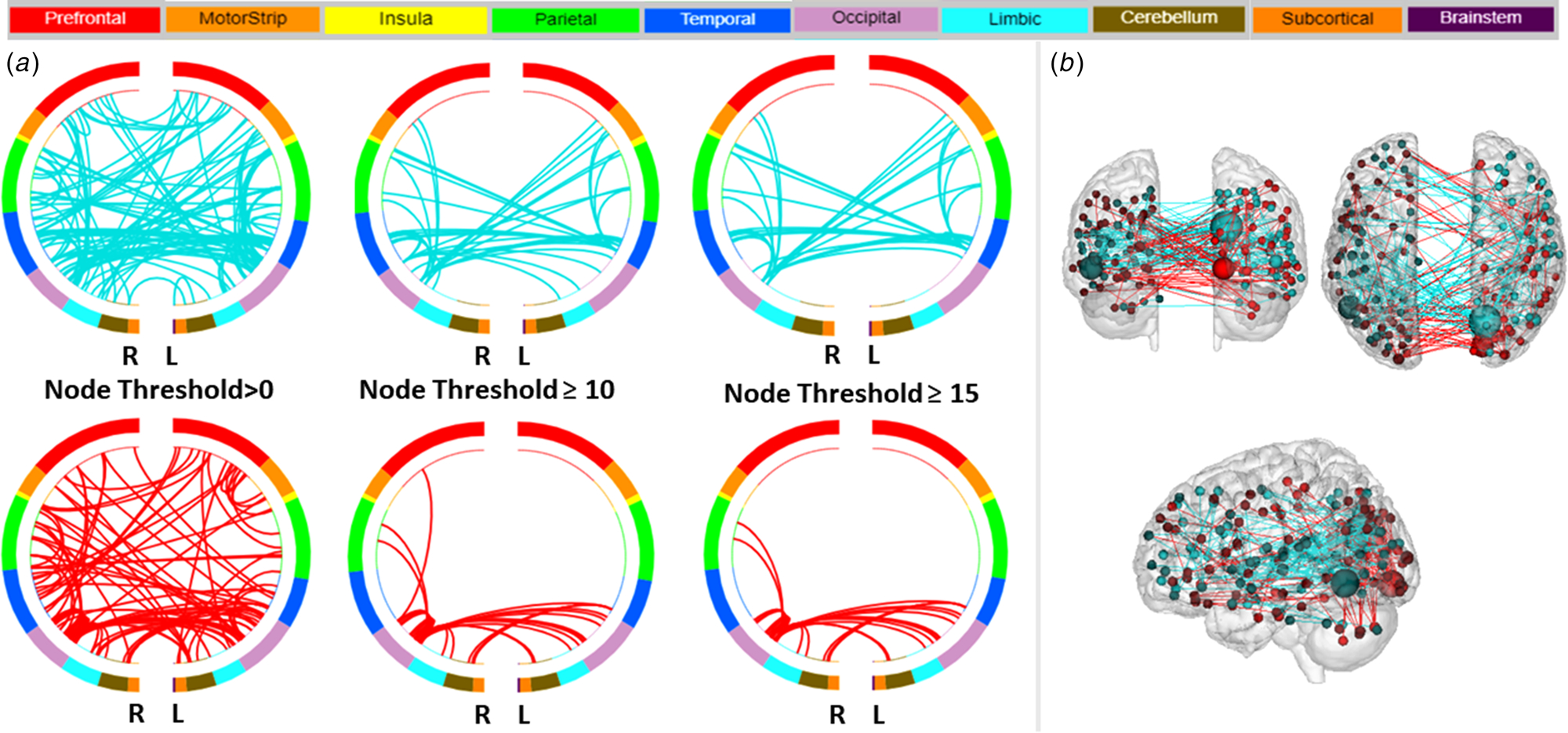
Negative and Positive Networks Predicting Severity of Elevated Mood using Connectome-based Predictive Modeling (CPM). (a) Circle plots in which nodes are assigned to one of ten bilateral macroscale brain regions. Negative and positive edges (or connections between nodes) are depicted on separate plots at different thresholds for visualization. Threshold values indicate the minimum number of connections emanating from a node. For the negative network (depicted in blue), decreased edge weights (i.e. decreased functional connectivity) predict severity of elevated mood, based on the Young Mania Rating Scale. For the positive network (in red), increased edge weights (i.e. increased functional connectivity) predict severity of elevated mood. R, right hemisphere; L, left hemisphere. (b) Glass brain depicting strength of negative and positive networks, depicted in blue and red respectively. Each node is represented as a sphere; the size of the sphere indicates the number of edges emanating from that node. The highest degree node, i.e., nodes with the most connections (edges), contributing to the prediction of depressed mood severity was a node located in the left fusiform gyrus in the negative network.

The overall CPM model also predicted the severity of elevated mood (YMRS) (combined positive and negative networks: *r* = 0.27, RMSE = 5.8, *p*_perm_ = 0.01; online Supplementary Fig. S2). Investigation of the positive and negative networks separately showed that the greater contribution for the prediction of YMRS scores was from the negative network (*r* = 0.25, RMSE = 5.9, *p*_perm_ = 0.02). Figure 2a, b display negative and positive YMRS networks, defined by the macroscale regions, that significantly predicted elevated mood severity. The high degree nodes in the negative network included a left temporal node located in the fusiform gyrus, and a right occipital node located in the visual association area. In predicting elevated mood severity, the left temporal node (fusiform) showed lower inter-hemispheric connections to occipital (right visual association area), lower intra-hemispheric connections to motor (left SMA), and lower interand intra-hemispheric connections to insular, temporal and parietal nodes. The right occipital node (visual association area) showed lower inter-hemispheric connections to occipital (left visual association area), insular, and motor (including left SMA) nodes, and lower intra-and inter-hemispheric connections to temporal (including left fusiform) and parietal nodes. The high degree node in the positive network that predicted the severity of elevated mood was the occipital lobe (right visual association area), which showed increased inter-hemispheric connection to occipital (left visual association area), increased intra-hemispheric connections to parietal, and increased inter- and intra-hemispheric connections to limbic (including right parahippocampal gyrus) nodes. The high degree nodes and their connections in the negative and positive network, and the respective macroscale regions they are assigned to, are detailed in Table 2. Sensitivity analysis when the model was also controlled for HDRS-5 showed the overall CPM model still predicted YMRS severity (combined positive and negative networks: *r* = 0.27, *p*_perm_ = 0.01); furthermore, the analysis showed no additional high degree nodes.

### Out-of-sample validation

The CPM prediction model generated by the training model predicted HDRS-5 (*r* = 0.45, df = 42, *p* = 0.002) and YMRS (*r* = 0.48, df = 40, *p* = 0.002) scores in the non-overlapping independent dataset. Prediction for HDRS-5 remained significant (*r* = 0.45, df = 40, *p* = 0.004) when the model was re-run after excluding the two subjects who did not have YMRS scores.

## Discussion

The study demonstrated that CPM, a recently developed connectome-based machine learning approach, predicted individual differences in severity of both depressed and elevated mood symptomatology in adults with BD using functional connectivity networks. The study, importantly, also demonstrated that these networks were predictive of mood symptomatology in an independent sample of adults with BD.

CPM parsed out distributed large scale functional connectivity networks that were predictive of depressed and elevated mood symptom severity. The negative and positive networks that contributed to depressed mood severity included dlPFC and SMA nodes with inter- and intra-hemispheric connections predominantly to other prefrontal, motor, limbic, temporo-parietal and cerebellar regions. No connections to nodes in the occipital cortex were observed from the high degree nodes in either the negative or the positive network. In contrast, the negative and positive networks that contributed to elevated mood severity included fusiform and visual association area high degree nodes with inter- and intra-hemispheric connections predominantly also to temporo-parietal, and motor regions, as well as to insula and occipital regions. Interestingly, unlike the ‘depressed mood’ network, no connections to nodes in the PFC or cerebellum were observed from the high degree nodes in the ‘elevated mood’ network. Investigation of the negative and positive networks separately showed that the greatest contribution for the prediction of both depressed and elevated mood was from their respective negative networks, and hence will be the focus of the discussion.

### Depressed mood severity

CPM showed decreased functional connectivity between the left dlPFC high degree node and the contralateral homologous region (i.e. right dlPFC) which constituted a part of the negative network that predicted depression severity (Table 1). The interhemispheric dysconnectivity in this region may suggest reduced functional coordination between both sides that could contribute to disturbances in the role of dlPFC in cognitive control and flexibility, especially over affective responses, a core feature in individuals with either unipolar or bipolar depression. Prior biological evidence for the role of dlPFC in depressed mood symptomatology has provided support for the use of this region as a target for stimulation by neuromodulation techniques for depression (Beynel et al., 2014; Kazemi et al., 2018; Leyman, De Raedt, Vanderhasselt, & Baeken, 2011; Tamas, Menkes, & El-Mallakh, 2007), although the interhemispheric observations extend beyond current models that focus on intra-hemispheric dlPFC connections.

Also predictive of depressed mood severity was reduced connectivity from the dlPFC to other anterior cortical (ventral and rostral PFC, ventral and dorsal ACC), posterior cortical (inferior temporal gyrus), and cerebellar regions that are implicated in various emotion regulation processes (Braunstein, Gross, & Ochsner, 2017; Ohira et al., 2006; Phillips, Ladouceur, & Drevets, 2008a; Schutter & van Honk, 2009; Wager, Davidson, Hughes, Lindquist, & Ochsner, 2008) (Table 1). This finding suggests that reduced top-down connectivity from the dlPFC to these regions may play a role in depressed mood severity in BD. Clinically, maladaptive regulation of emotions has been shown to play a major role in the pathophysiology of depression (Abravanel & Sinha, 2015). In particular, emotion dysregulation tends to worsen mood, contribute to feelings of excessive guilt, reduced motivated behavior, and be a risk factor for suicidal thoughts and behaviors – all features captured by the items in the HDRS-5 scale.

Despite psychomotor retardation being a common feature during the depressed state of BD, very few studies have focused on the involvement of motor areas in the pathophysiology of the disorder. Although speculative, the lower connectivity observed between the SMA and temporoparietal and primary motor regions might help to explain psychomotor abnormalities of BD depression. The present study also showed lower SMA-amygdala connectivity that was predictive of depressed mood severity. Direct structural connections between the amygdala and the motor region have been observed in animal models (Grèzes, Valabrègue, Gholipour, & Chevallier, 2014) which may suggest a mechanism by which aberrant signals from the amygdala may influence complex motor behavior in BD depression. Further in-depth studies are required to confirm the involvement of SMA in the psychomotor abnormalities of BD depression. Notwithstanding, the pattern of connections in the ‘depressed mood symptom network’ suggests that disruptions in brain networks associated with cognitive control and flexibility, emotion regulation, and psychomotor functioning predict depressed mood severity.

### Elevated mood severity

The present study showed that more posterior cortical regions, i.e., the left fusiform and the right visual association area comprised the high degree nodes that predicted elevated mood severity. While the fusiform and the visual association are both important for processing visual stimuli, the fusiform has specialized roles in recognizing faces as well as facial expressions (Ganel, Valyear, Goshen-Gottstein, & Goodale, 2005; Hadjikhani & de Gelder, 2003). Decreased connectivity between the fusiform and brain areas such as the insula and somatosensory areas, which are regions important in the detection of salient stimuli (Uddin, 2015) and social perceptual processes, respectively, may be suggestive of the involvement of brain networks that underlie impairments in recognizing salient social cues in elevated mood states. Decreased connectivity was also observed between the fusiform gyrus and motor areas (primary motor and SMA), regions that are involved in motor behavior and are influenced by visual cues (Oliveri et al., 2003; Rodigari & Oliveri, 2014). Although speculative, the pattern of connections in the ‘elevated mood symptom network’ implies deficits in social perceptual functions which may contribute to social disinhibition, high levels of motor activity, and other disinhibited behavior typically observed in individuals with elevated mood. The dysconnectivity between the visual face processing and salient network nodes in the prediction of elevated mood severity suggests that the emotional face paradigm may have helped to amplify individual differences in predictive networks in the present study. Future work with emotional facial and non-facial stimuli is, however, required to confirm this theory.

The above findings should be considered preliminary as further testing using larger samples (~1000) is needed to reduce the risk of machine learning performance misestimation (Flint et al., 2021), and as the predictive networks were tested in an independent sample of fewer than 50 individuals. Furthermore, the concordance rates between the actual and predicted mood severity scores in our study were modest. This may be because the clinical and underlying neurobiological heterogeneity in the sample is not adequately captured by the mood rating scales. The concordance rates, however, are in line with previous studies using CPM to predict individual behaviors (Ju et al., 2020; Lin et al., 2022; Rutherford, Potenza, Mayes, & Scheinost, 2020; Wang et al., 2021).The aforementioned clinical heterogeneity in the sample is another limitation of the study. This sample included participants who were either BD-I or BD-II, in different phases of their illness (including mixed states), and had varied psychiatric comorbidities. For instance, deficits in brain regions subserving social perceptual functions observed in relation to elevated mood symptom severity have previously been observed in individuals with post-traumatic stress disorder (PTSD) (Stevens & Jovanovic, 2019), and 20% of the current sample had comorbid PTSD. Two-thirds of the BD participants were on different combinations of psychiatric medications. There have been reports of effects of psychiatric medications on the brain regions involved in this study. Prior reviews support the view that psychotropic medications have a normalizing effect on brain function, rather than brain differences observed in BD being the result of psychotropic medications (Hafeman, Chang, Garrett, Sanders, & Phillips, 2012; Phillips, Travis, Fagiolini, & Kupfer, 2008b). Lastly, the predictive networks may be specific to the HDRS-5 and YMRS scales that were used to measure depressed and elevated mood symptom severity in this study. For example, the cerebellum appeared as a high degree node in the analyses of depression severity, when the HDRS 29-item version was used.

## Conclusions

The study demonstrates that distributed connectivity networks could be identified using CPM that predicted individual differences in depressed and elevated mood severity in adults with BD. Importantly, these networks were predictive of mood symptomatology in an independent and heterogenous sample of adults with BD. Data demonstrate that connectivity differences in networks subserving emotion regulation, cognitive, and psychomotor function predict depressed mood severity, while connectivity differences in networks subserving social perceptual functions predict elevated mood severity. These connectome fingerprints may be biomarkers for targeted therapeutic approaches to reduce depressed and elevated mood symptoms in individuals with BD.

## Supplementary Material

Supplement

## Figures and Tables

**Table 1.** Highest degree nodes and their connections in the positive and negative networks predictive of depressed mood severity in adults with bipolar disorder

Network	Node	Connections						Motor
		Prefrontal	Insula	Limbic	Temporal	Parietal	Cerebellum	
Negative	L Prefrontal (L dlPFC)	R dlPFC	–	L vACC	L ITG	–	L/R Cerebellum	–
		L medial OFC		L dACC				
		L/R rostral PFC						
	L Motor (SMA)	–	–	R Amyg.	L STG	L/R SMG	–	L/R Pri. Motor
			–	L dPCC				
Positive	L Motor (SMA)	L dlPFC	–	–	L/R Fusiform	L Angular Gyrus	R Cerebellum	–
		L rostral PFC						
		L Fr. Eye Field						
	L Prefrontal (L dlPFC)	R vlPFC	R Insula	R vACC	–	L/R SMG	–	L SMA
				R dPCC				

Abbreviations: L, Left Hemisphere; R, Right Hemisphere; L/R, Bilateral; PFC, Prefrontal Cortex; OFC, Orbitofrontal Cortex; dlPFC, Dorsolateral Prefrontal Cortex; vlPFC, Ventrolateral Prefrontal Cortex; vACC, Ventral Anterior Cingulate Cortex; dACC, Dorsal Anterior Cingulate Cortex; dPCC, Dorsal Posterior Cingulate Cortex; Amg, Amygdala; Fr. Eye Field, Frontal Eye Field; SMG, Supramarginal Gyrus, STG, Superior Temporal Gyrus; ITG, Inferior Temporal Gyrus; SMA, Supplementary Motor Area; Pri. Motor, Primary Motor.

**Table 2.** Highest degree nodes and their connections in the positive and negative networks predictive of elevated mood severity in adults with bipolar disorder

Network	Node	Connections						Occipital	Motor
		Prefrontal	Insula	Limbic	Temporal	Parietal	Cerebellum		
Negative	L Temporal (Fusiform)	–	L/R Insula	–	L/R STG	L/R SMG	–	R Visual Ass.	L SMA
					R ITG	R Pri. Sensory			
	R Occipital (Visual Ass.)	–	L Insula	–	L/R STG	L SMG	–	L Visual Ass.	L SMA
					L Fusiform	R Pri. Sensory			L Pri. Motor
Positive	R Occipital (Visual Ass.)	–	–	R dPCC	–	R SMG	–	L Visual Ass.	–
				R Parahipp.					
				L vPCC					

Abbreviations: L, Left Hemisphere; R, Right Hemisphere; L/R, Bilateral; vPCC, Ventral Posterior Cingulate Cortex; dPCC, Dorsal Posterior Cingulate Cortex; Parahipp, Parahippocampal Gyrus; SMG, Supramarginal Gyrus, STG, Superior Temporal Gyrus; ITG, Inferior Temporal Gyrus; SMA, Supplementary Motor Area; Pri. Motor, Primary Motor; Pri. Sensory, Primary Somato-Sensory Area; Visual Ass., Visual Association Area.
