# Supplementary material for: Predicting depressed and elevated mood symptomatology in bipolar disorder using brain functional connectomes
Source: Psychol Med. Author manuscript; Available in PMC 2023 Oct 27. (PMC10491744; doi:10.1017/S003329172300003X)
Supplement: Supplement [file NIHMS1907688-supplement-Supplement.docx]

**
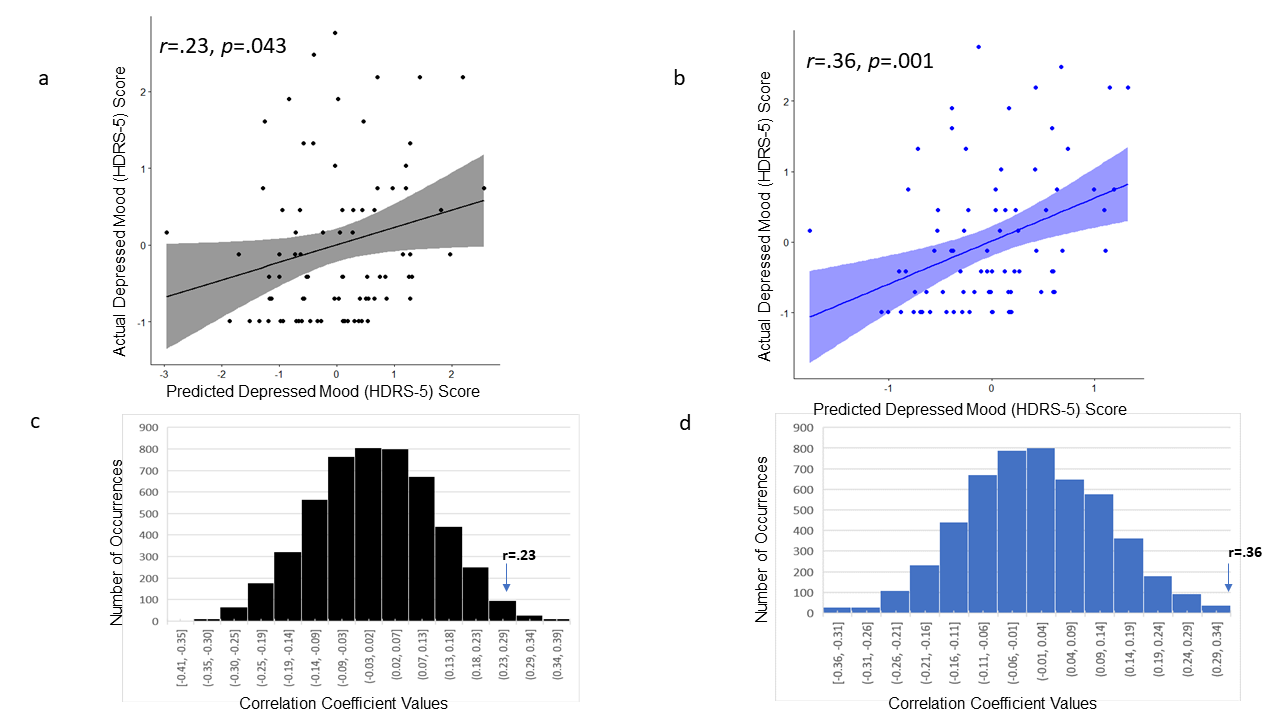
SUPPLEMENTAL MATERIAL**

**Figure S1: Connectome-based Model Performance for Predicting Depressed Mood Severity**

The graphs show the correlations between predicted (x-axis) and actual (y-axis) severity scores of depressed mood (five items on the Hamilton Depression Rating Scale, HDRS-5) in the (a) combined and (b) negative networks generated using Connectome-based Predictive Modeling (CPM). Predicted and actual severity scores are standardized to z-scores and the correlation between the scores (*r*) and the corresponding significance value (*p*) are presented. The distribution of correlations with a permutation test of 5000 times in the (c) combined and (d) negative networks.

**
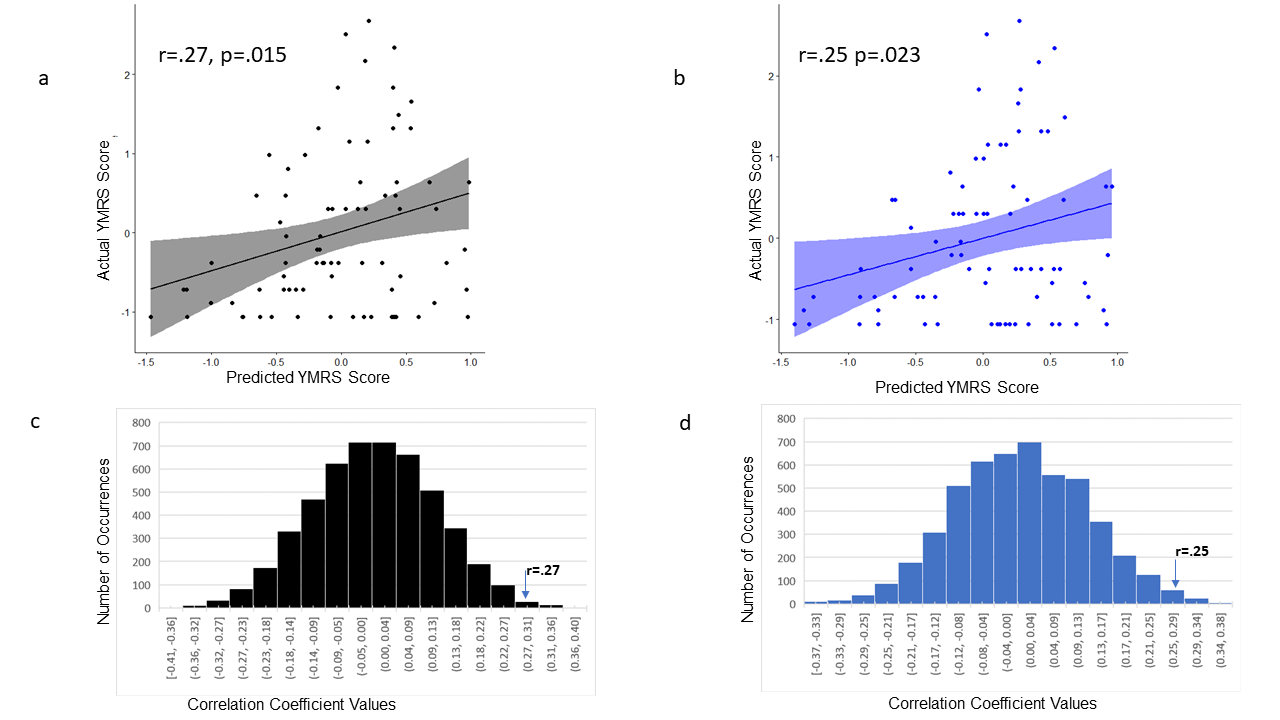
**

**Figure S2: Connectome-based Model Performance for Predicting Elevated Mood Severity**

The graphs show the correlations between predicted (x-axis) and actual (y-axis) severity scores of elevated mood (Young Mania Rating Scale, YMRS, scores) in the combined (a) and negative (b) networks generated using Connectome-based Predictive Modeling. Predicted and actual severity scores are standardized to z-scores and the correlation between the scores (*r*) and the corresponding significance value (*p*) are presented. The distribution of correlation by a permutation test of 5000 times in the (c) combined and (d) negative networks.

**TABLE S1. Participant Demographic and Clinical Features**

| **Characteristics Participants with BD** | | | | |  |  |  |
| --- | --- | --- | --- | --- | --- | --- | --- |
|  | **Mean** | | | **SD** |  |  |  |
| Age (years) | 29.3 | | | 11.1 |  |  |  |
| HDRS-29 | 12.3 | | | 10.1 |  |  |  |
| HDRS-5 | 3.4 | | | 3.4 |  |  |  |
| YMRS | 6.5 | | | 6.1 |  |  |  |
|  | **N** | | | **%** |  |  |  |
| Females | 51.0 | | | 63 |  |  |  |
| Mood state at scan: | |  |  | | | | |
| Depressed | | 27.0 | 33.3 | | |  |  |
| Euthymic | | 26.0 | 32.1 | | |  |  |
| Elevated | | 15.0 | 18.5 | | |  |  |
| Mixed | | 13.0 | 16.1 | | |  |  |
| BDI (vs BDII) | | 71.0 | 87.7 | | |  |  |
| Rapid cycling^a^ | | 41.0 | 50.6 | | |  |  |
| Lifetime psychosis | | 29.0 | 35.8 | | |  |  |
| Unmedicated at scan | | 27.0 | 33.3 | | |  |  |
| Lifetime medications^a^ | |  |  | | |  |  |
| Anticonvulsants | | 53.0 | 66.3 | | |  |  |
| Antipsychotics | | 59.0 | 73.8 | | |  |  |
| Antidepressants | | 62.0 | 77.5 | | |  |  |
| Lithium carbonate | | 37.0 | 48.1 | | |  |  |
| *Comorbidity* | |  |  | | |  |  |
| Lifetime substance use disorders | |  |  | | |  |  |
| Alcohol dependence | | 11.0 | 13.6 | | |  |  |
| Alcohol abuse | | 14.0 | 17.3 | | |  |  |
| Cannabis dependence | | 11.0 | 13.6 | | |  |  |
| Cannabis abuse | | 9.0 | 11.1 | | |  |  |
| Cocaine dependence | | 8.0 | 9.9 | | |  |  |
| Cocaine abuse | | 3.0 | 3.7 | | |  |  |
| Opiate dependence | | 4.0 | 4.9 | | |  |  |
| Opiate abuse | | 1.0 | 1.2 | | |  |  |
| Polysubstance dependence | | 3.0 | 3.7 | | |  |  |
| Polysubstance abuse | | 1.0 | 1.2 | | |  |  |
| Lifetime other psychiatric disorders^a^ | |  |  | | | |  |
| Post-traumatic stress disorder | | 16.0 | 20.0 | | |  |  |
| Panic disorder | | 11.0 | 13.8 | | |  |  |
| Social phobia | | 9.0 | 11.3 | | |  |  |
| Generalized anxiety disorder | | 7.0 | 8.8 | | |  |  |
| Specific phobia | | 8.0 | 10.0 | | |  |  |
| Obsessive-compulsive disorder | | 4.0 | 5.0 | | |  |  |
| Anorexia nervosa | | 6.0 | 7.5 | | |  |  |
| Bulimia nervosa | | 5.0 | 6.3 | | |  |  |
| Binge eating disorder | | 3.0 | 3.8 | | |  |  |
| Eating Disorder NOS | | 2.0 | 2.5 | | |  |  |

Abbreviations: HDRS-29: 29-item Hamilton Depression Rating Scale; YMRS: Young Mania Rating Scale; HDRS-5: 5-item Hamilton Depression Rating Scale (summing five items from the HDRS that showed the highest loading for depression (i.e., depressed mood, work and interests, guilt, psychomotor retardation, and suicide); YMRS: Young Mania Rating Scale; NOS -Not Otherwise Specified; SD: Standard Deviation; N: Number of cases; %: Percentage of cases. ^a^Scores not available: Rapid cycling, lifetime anti-convulsant, anti-depressant, antipsychotic use, and lifetime other psychiatric disorders comorbidity for one participant, and lifetime lithium carbonate use for four participants.

**TABLE S2: High degree nodes and their connections in the negative and positive networks predictive of the 29-item Hamilton Depression Rating Scale severity in adults with bipolar disorder**

| **NETWORK** | **NODE** |  | | **CONNECTIONS** | | | | | | |  |  |
| --- | --- | --- | --- | --- | --- | --- | --- | --- | --- | --- | --- | --- |
|  |  | **Prefrontal** | | | **Subcortical** | **Insula** | **Limbic** | **Temporal** | **Parietal** | **Cerebellum** | **Motor** | **Occipital** |
| Negative | L Prefrontal | R dlPFC | |  |  | - | L vACC | L ITG | - | L/R Cerebellum | - | - |
|  | (L dlPFC) |  |  |  |  |  |  |  |  |  |  |  |
|  |  | L medial OFC | | |  |  | L dACC |  |  |  |  |  |
|  |  | L/R rostral PFC | | |  |  |  |  |  |  |  |  |
|  |  |  |  |  |  |  |  |  |  |  |  |  |
|  | R Prefrontal | L dlPFC | | | R Caudate | - | R vACC | - | - | - | - | - |
|  | (R dlPFC) |  |  |  |  |  |  |  |  |  |  |  |
|  |  | R medial OFC | | |  |  | L dACC |  |  |  |  |  |
|  |  | L/R rostral PFC | | |  |  |  |  |  |  |  |  |
|  |  | L Fr. Eye Field | | |  |  |  |  |  |  |  |  |
|  |  | L IFG | | |  |  |  |  |  |  |  |  |
|  |  |  |  |  |  |  |  |  |  |  |  |  |
|  |  |  |  |  |  |  |  |  |  |  |  |  |
| Positive | R Cerebellum | L medial OFC | | |  | R Insula | R vACC | R STG | L/R Pri. Sensory | R Cerebellum | L/R Pri. Motor | L/R Visual Ass. |
|  |  |  |  |  |  |  |  | R Pri. Auditory |  |  |  |  |

Abbreviations: L: Left Hemisphere; R: Right Hemisphere; L/R: Bilateral; PFC: Prefrontal Cortex; OFC: Orbitofrontal cortex; dlPFC: Dorsolateral Prefrontal Cortex; vlPFC: Ventrolateral Prefrontal Cortex; vACC: Ventral Anterior Cingulate Cortex; dACC: Dorsal Anterior Cingulate Cortex; STG: Superior Temporal Gyrus; ITG: Inferior Temporal Gyrus; IFG: Inferior Frontal Gyrus; Fr. Eye Field: Frontal Eye Field; Prim. Auditory: Primary Auditory; Pri. Sensory: Primary Sensory; Pri. Motor: Primary Motor; Visual Ass: Visual Association Area
